# Pugilism among Irish Travelers: cultural tradition and the fight bite injury

**DOI:** 10.5249/jivr.v8i1.673

**Published:** 2016-01

**Authors:** Toral R. Patel, Anand N. Bosmia, R. Shane Tubbs

**Affiliations:** ^*a*^Children’s of Alabama – Pediatric Neurosurgery, Birmingham,USA.

The extant medical literature lacks a discussion of the cultural tradition of bare-knuckle boxing among Irish Travelers, which is pertinent to traumatology given the possible medical complications of this practice. This cultural tradition is a risk factor for a potentially dangerous injury known as a “fight bite”. Irish Travelers are individuals belonging to nomadic families who originated in Ireland several centuries ago, and bare-knuckle boxing is a means by which a male Irish Traveler upholds his family’s honor, as shown in the 2011 documentary film Knuckle.^1^ Rival families or clans may hold grudges against one another for decades, and in response to taunts from a rival clan, which are sometimes posted on video-sharing websites such as YouTube, male Irish Travelers compete with each other in bare-knuckle boxing matches to settle the score.^2^

A fight bite is a clenched fist injury that occurs when one person strikes another person’s mouth or when a fist is bitten directly, and such an injury can inoculate the site of penetration with bacteria or other infectious agents found in the human mouth.^3^ However, in the case of bare-knuckle boxing matches among Irish Travelers, a fighter’s fist is less likely to be bitten because the matches are overseen by referees. Punch or tooth-puncture wounds generally produce a laceration over the third, fourth, or fifth dorsal metacarpophalangeal (MCP) joint, and after contact is made, the victim likely will extend his hand and thereby deeply inoculate the infectious agent via the extensor tendon and soft tissue.^3^ The classic injury consists of penetration by a tooth of the dorsum of the hand over the third MCP joint.^3^ Patients who sustain fight bites may develop an infection at the site of penetration, and some patients even become septic and must be hospitalized.^4^ Permanent disability or morbidity can result if the patient is not given appropriate antibiotics,^4^ as persistent infection and ensuing necrosis of the injured limb may necessitate amputation of that limb.

Decreased health-seeking behavior is reported among male Irish Travelers, which is partly due to the belief that illness signifies weakness.^5^ Thus, male Irish Travelers who participate in bare-knuckle boxing are at risk for complications from fight bites secondary to delayed care or failure to seek medical care, although resistance towards treatment with antibiotics is less likely among male Irish Travelers who actively seek medical care for traumatic injuries caused by bare-knuckle boxing. Healthcare professionals should be aware of this pugilistic tradition among the Irish Traveler community and the high probability that these patients will not be dissuaded easily from participating in bare-knuckle boxing in the future.
